# Pathogenic Mechanisms of Pulmonary Arterial Hypertension: Homeostasis Imbalance of Endothelium-Derived Relaxing and Contracting Factors

**DOI:** 10.1016/j.jacasi.2022.09.010

**Published:** 2022-12-15

**Authors:** Jinsheng Zhu, Lei Yang, Yangfan Jia, Angela Balistrieri, Dustin R. Fraidenburg, Jian Wang, Haiyang Tang, Jason X-J Yuan

**Affiliations:** a State Key Laboratory of Respiratory Disease, National Clinical Research Center for Respiratory Disease, Guangdong Key Laboratory of Vascular Disease, Guangzhou Institute of Respiratory Health, The First Affiliated Hospital of Guangzhou Medical University, Guangzhou, China; b College of Veterinary Medicine, Northwest A&F University, Yangling, China; c Section of Physiology, Division of Pulmonary, Critical Care and Sleep Medicine, Department of Medicine, University of California, San Diego, La Jolla, California, USA; d Division of Pulmonary, Critical Care, Sleep, and Allergy, Department of Medicine, University of Illinois at Chicago, Chicago, Illinois, USA

**Keywords:** endothelial dysfunction, endothelium-derived relaxing factor, pulmonary arterial hypertension, vascular homeostasis, 5-HT, 5-hydroxytryptamine, ACE, angiotensin-converting enzyme, cGMP, cyclic guanosine monophosphate, EC, endothelial cell, EDCF, endothelium-derived contracting factor, EDRF, endothelium-derived relaxing factor, ET, endothelin, PAH, pulmonary arterial hypertension, PASMC, pulmonary artery smooth muscle cell, PG, prostaglandin, TPH, tryptophan hydroxylase, TXA_2_, thromboxane A_2_

## Abstract

Pulmonary arterial hypertension (PAH) is a progressive and fatal disease. Sustained pulmonary vasoconstriction and concentric pulmonary vascular remodeling contribute to the elevated pulmonary vascular resistance and pulmonary artery pressure in PAH. Endothelial cells regulate vascular tension by producing endothelium-derived relaxing factors (EDRFs) and endothelium-derived contracting factors (EDCFs). Homeostasis of EDRF and EDCF production has been identified as a marker of the endothelium integrity. Impaired synthesis or release of EDRFs induces persistent vascular contraction and pulmonary artery remodeling, which subsequently leads to the development and progression of PAH. In this review, the authors summarize how EDRFs and EDCFs affect pulmonary vascular homeostasis, with special attention to the recently published novel mechanisms related to endothelial dysfunction in PAH and drugs associated with EDRFs and EDCFs.

## Highlights

• Disrupted EDRF/EDCF homeostasis contributes to the development and progression of PAH/PH.
• Understanding EDRF/ECRF interaction helps in developing therapeutic interventions for PAH/PH.
• Multiple trials are undergoing for drugs based on EDRF/ECRF mechanisms for PAH/PH therapy.

Endothelial dysfunction, characterized by structural changes and functional impairment of the pulmonary artery, plays an important role in the development and progression of pulmonary arterial hypertension (PAH). Normal synthesis and release of endothelium-derived relaxing factors (EDRFs) is considered a sign of endothelial integrity. EDRFs mainly include nitric oxide (NO), prostacyclin, and endothelium-derived hyperpolarizing factors.^1^ Under the pathologic conditions of PAH, decreased EDRFs, increased endothelium-derived contracting factors (EDCFs) and increased sensitivity of EDCF receptors in endothelial cells (ECs) are the direct cause of endothelial dysfunction.^2^ Increased EDCFs, including endothelin (ET)-1, EC-derived adenosine diphosphate, angiotensin II, 5-hydroxytryptamine (5-HT), 8-iso-prostaglandin F_2α_ (PGF_2α_), and thromboxane A_2_ (TXA_2_), which can also antagonize the protective and vasodilative effects of EDRFs, ultimately lead to endothelial dysfunction. The chemical properties and classification of these factors are described in detail (Table 1).

**Table 1:** Physicochemical Properties and Classification of Endothelium-Derived Factors

Factor	Property	Classification
NO	Inorganic gases	EDRF
PGI_2_	Arachidonic acid metabolites	EDRF
H_2_S	Inorganic gases	EDRF, EDHF
K^+^	Ion	EDRF, EDHF
EETs	Arachidonic acid metabolites	EDRF, EDHF
Ang-(1-7)	Bioactive peptide	EDRF
Adenosine	Nucleoside	EDRF
EC-derived ATP	Nucleotide	EDRF
ROS	Oxygen radical	EDCF
EC-derived ADP	Nucleotide	EDCF
EC-derived Up4A	Nucleotide	EDCF
ET-1	Bioactive peptide	EDCF
5-HT	Small organic molecule	EDCF
TXA_2_	Arachidonic acid metabolites	EDCF
8-iso-PGF_2α_	Arachidonic acid metabolites	EDCF
Ang II	Bioactive peptide	EDCF

5-HT = 5-hydroxytryptamine; Ang = angiotensin; EC = endothelial cell; EDCF = endothelium-derived contracting factor; EDHF = endothelium-derived hyperpolarizing factor; EDRF = endothelium-derived relaxing factor; EET = epoxyeicosatrienoic acid; ET-1 = endothelin 1; PG = prostaglandin; ROS = reactive oxygen species; TXA_2_ = thromboxane A_2_; Up4A = uridine adenosine tetraphosphate.

It is widely accepted that endothelial dysfunction and apoptosis is critical in the initiation of PAH. EC apoptosis during disease initiation activates a highly proliferating population of pathogenic ECs, which drive PAH progression.^3^ Broadly, endothelial dysfunction is considered to be a complex set of biological processes involving EDRF/EDCF imbalance, inflammatory cell adhesion, platelet aggregation, increased oxidative stress and glycolysis, endothelial-to-mesenchymal transition, and others.^4^

Pulmonary artery smooth muscle cells (PASMCs) and pulmonary artery ECs, as direct participants in pulmonary vascular remodeling, have been the main focus of study in the field of PAH.^5^ For PASMCs, proliferation, migration, and media hyperplasia are thought to contribute significantly to pulmonary artery remodeling.^6^ For pulmonary artery ECs, endothelial dysfunction is one of the typical vascular alterations in the development of PAH.^7^ In this review, we discuss the role of EDRFs and EDCFs in endothelial function during PAH progression. On the basis of recent findings, we summarize new mechanisms of endothelial dysfunction and novel related targeted drugs in PAH clinical therapy.

## Role of Edcfs and Edrfs in PAH Pathology

### Nitric oxide

NO, as a vasodilator, is produced by 2 pathways: the classical L-arginine-to-NO pathway and the nonclassical nitrate-nitrite-to-NO pathway.^8^ In the classic pathway, the pulmonary vasculature primarily uses NO produced by converting L-arginine through NO synthase. The endothelial NO synthase-mediated biosynthesis of NO in ECs is considered to be the main source of bioavailable NO in the pulmonary circulation.^9^ Endogenous NO inhibits apoptosis and promotes cell proliferation by promoting the expression of vascular endothelial growth factor, which is essential for angiogenesis in pulmonary vascular development.^10^ Moreover, NO also can be released into adjacent PASMCs to convert guanosine triphosphate to cyclic guanosine monophosphate (cGMP) by interacting with soluble guanylate cyclase. cGMP achieves its function by activating the downstream cGMP-dependent protein kinase G, cGMP-gated cation channels, and phosphodiesterases. Protein kinase G regulates intracellular calcium [Ca^2+^]_i_ concentrations by affecting several cytosolic Ca^2+^ flux regulators to relax vascular tension.

NO synthesis via endothelial NO synthase depends on the availability of substrates and cofactors. Sufficient tetrahydrobiopurine and L-arginine are essential for the maintenance of NO synthesis by endothelial NO synthase. Considerable evidence indicates that L-arginine and tetrahydrobiopurine bioavailability is significantly reduced in pulmonary vascular diseases with endothelial dysfunction.^11^^,^^12^ The L-arginine antagonists asymmetrical dimethylarginine and symmetrical dimethylarginine were remarkably increased in the plasma and tissues of both rats with pulmonary hypertension and patients with idiopathic PAH.^13^ Asymmetrical dimethylarginine inhibits endothelial NO synthase activity through direct binding, which leads to endothelial NO synthase uncoupling and superoxide accumulation.^14^ Low tetrahydrobiopurine levels or excess oxidized biopterin (dihydrobiopterin) cause endothelial NO synthase uncoupling and the reduction of oxygen to a superoxide anion. This then scavenges NO and generates other reactive oxygen species, resulting in constrictive and proliferative vascular pathology.^15^^,^^16^

The progression of PAH is significantly associated with a reduction in endothelial NO synthase expression, which is what may contribute to pulmonary vasoconstriction and media hypertrophy.^17^ In PAH, low endothelial NO synthase levels in pulmonary vascular ECs impair NO production, which may lead to increased vascular tone and other cellular activity in the vascular wall.^18^ Interestingly, recent studies have shown that the protein expression of endothelial NO synthase did not change in experimental pulmonary hypertension. However, endothelial NO synthase uncoupling leads to its functional loss and an increase in reactive oxygen species production.^19^ Furthermore, NO is further inactivated by interactions with reactive oxygen species, which results in a reduction in available NO for vasodilation and antiproliferation.

### Prostaglandins and their receptors

**Figure 1:**
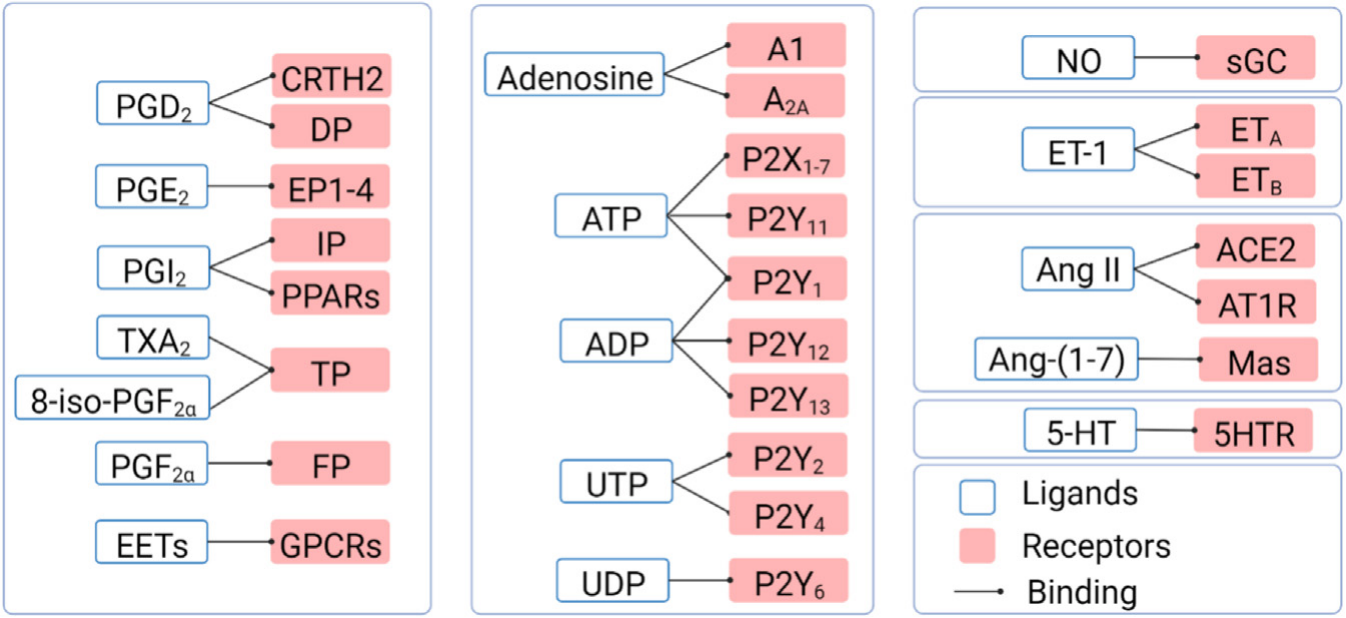
Endothelium-Derived Factors and Their Receptors The regulatory effect of endothelium-derived relaxing factors (EDRFs) and endothelium-derived contracting factors (EDCFs) on vascular tension depends on their specific receptor pathways. 5-HT = 5-hydroxytryptamine; 5HTR = 5-hydroxytryptamine receptor; 8-iso-PGF_2α_ = 8-iso-prostaglandin F_2α_; A1, A_2A_ = type 1 purinergic receptors; ACE2 = angiotensin-converting enzyme 2; ADP = adenosine diphosphate; Ang = angiotensin; AT1R = angiotensin type 1 receptor; ATP = adenosine triphosphate; CRTH2 = prostaglandin D_2_ receptor 2; DP = prostaglandin D_2_ receptor; EET = epoxyeicosatrienoic acid; EP = prostaglandin E receptor; ET = endothelin; ET_A_ = endothelin receptor A; ET_B_ = endothelin receptor B; FP = prostaglandin F_2α_ receptor; GPCR = G protein–coupled receptor; IP = prostaglandin I_2_ receptor; Mas = Mas receptor; P2X = type 2X purinergic receptor; P2Y = type 2Y purinergic receptor; PG = prostaglandin; sGC = soluble guanylate cyclase; TP = thromboxane A_2_ receptor; TXA_2_ = thromboxane A_2_; UDP = uridine diphosphate; UTP = uridine triphosphate.

Arachidonic acid is catalyzed by cyclooxygenases and PGI_2_ synthetases to produce a series of prostaglandins (PGs). The binding relationship between these ligands and receptors^20^ and their functions are shown in Figure 1. PGD_2_ effectively increased pulmonary blood flow and reduced pulmonary vascular resistance and pulmonary artery pressure in newborn lambs with pulmonary hypertension.^21^ However, intravenous PGD_2_ injection failed to improve hemodynamic parameters and oxygenation in newborn human infants with persistent pulmonary hypertension of the newborn.^22^ Two subsequent critical clinical studies suggested the importance of the PGD_2_ signaling pathway in pulmonary hypertension. It was found that the concentration of eicosanoid (including PGD_2_) increased in the bronchoalveolar lavage fluid of patients with persistent pulmonary hypertension of the newborn.^23^ In contrast, concentrations of the TXA_2_ and PGD_2_ metabolites TX-M and PGD-M simultaneously increased in the urine of patients with primary pulmonary hypertension.^24^ The PGD_2_ receptor CRTH2 is up-regulated in circulating T helper type 2 cells in patients with idiopathic PAH and in rodent pulmonary hypertension models, while T helper type 2 cell–specific CRTH2 knockout alleviated pulmonary hypertension in rodents.^25^ Similarly, macrophage-derived PGD_2_ dilated blood vessels via PGD_2_ receptor 1 on PASMCs.^26^ PGD_2_ recruits T helper type 2 cells to form a local inflammatory microenvironment through the CRTH2 receptor. In contrast, it can dilate blood vessels through PGD_2_ receptor 1.

PGE_2_ is a widely expressed lipid signaling molecule involved in pain, vascular tension regulation, tissue damage repair, and inflammatory response.^27^ PGE_2_ receptors 1, 2, 3, and 4 generally exist on smooth muscle cells. PGE_2_ receptor 1 activation stimulates intracellular calcium and promotes vasoconstriction. The activation of PGE_2_ receptors 2 and 4 stimulates the cyclic adenosine monophosphate–protein kinase A signaling pathway to promote vasodilation, while PGE_2_ receptor 3 inhibits vasodilation in the opposite way.^28^ PGE_2_ signaling usually causes airway smooth muscle relaxation. Studies have shown that activation of airway PGE_2_ receptor 4 had additional benefits for group III pulmonary hypertension treatment,^29^ while endothelial-specific knockout of PGE_2_ receptor 4 impaired NO synthesis.^30^

**Figure 2:**
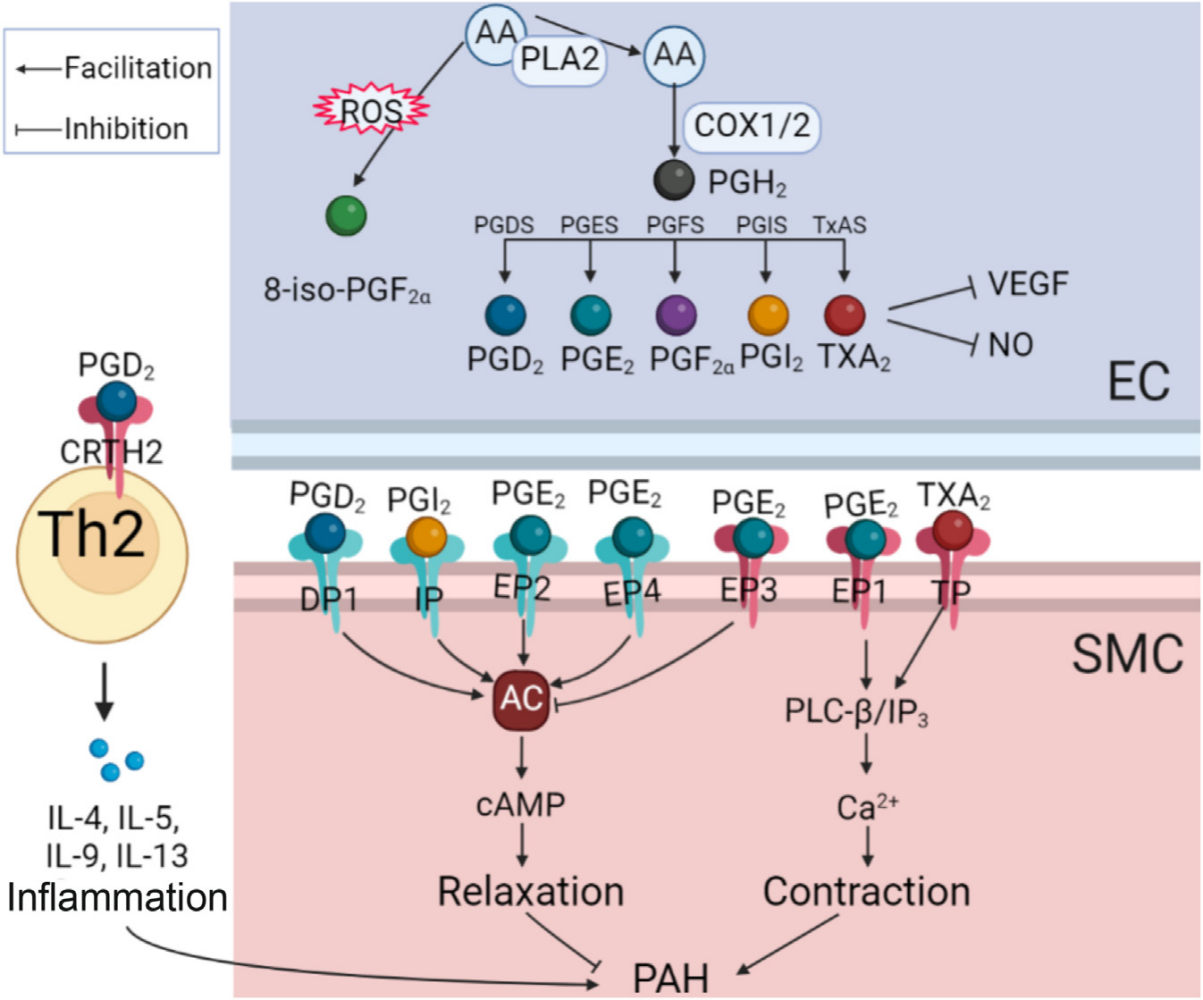
Prostaglandin Signaling Regulates Vascular Tone in PAH Progression PGI_2_, PGD_2_, and PGE_2_ relax smooth muscle cells (SMCs) through IP, PGD_2_ receptor 1, and EP2 and EP4 receptors, respectively. PGE_2_ constricts blood vessels through EP1 and EP3. PGD_2_ promotes inflammation and PAH through CRTH2 of T helper type 2 (Th2) cells. AA = arachidonic acid; AC = adenylate cyclase; cAMP = adenosine cyclic phosphate; COX1/2 = cyclooxygenase 1/2; EC = endothelial cell; IL = interleukin; IP_3_ = inositol triphosphate; PGDS = prostaglandin D synthase; PGES = prostaglandin E synthase; PGFS = prostaglandin F synthase; PGIS = prostaglandin I synthase; PL = phospholipase; ROS = reactive oxygen species; TxAS = thromboxane synthase; VEGF = vascular endothelial growth factor; other abbreviations as in Figure 1.

PGF_2α_ appears to contribute to the development of pulmonary hypertension. Earlier case reports suggested that PGF_2α_ metabolism was impaired in the context of extensive pulmonary vascular injury.^31^ PGF_2α_ and its receptors usually play a role in the reproductive system and renal function, and there is evidence that PGF_2α_ promotes cardiomyocyte hypertrophy in vivo.^32^ In vitro experiments in the pulmonary arteries of rats demonstrated that PGF_2α_ enhanced pulmonary vasoconstriction under euhydric hypercapnic conditions.^33^ Hypoxia caused by pulmonary diseases can alter angiogenesis, metabolism, and apoptosis by activating the expression of hypoxia-inducible factors to impair EC function.^34^ The signaling pathways of PGs in regulating vascular tone are shown in Figure 2.

In addition to the effects on pulmonary artery ECs and PASMCs, PGD_2_, PGE_2_, and PGI_2_ all increased intracellular cyclic adenosine monophosphate levels to promote apoptosis of fibroblasts and inhibit cell proliferation and transformation. Antifibrotic effects of PGI_2_ have been reported in dog cardiac hypertrophy models since the 1980s.^35^ In gamma delta T cells, PGD_2_ can activate CRTH2 receptors and promote the release of interleukin-10 to inhibit fibrosis.^36^ PGE_2_ also plays an antifibrotic role, primarily through binding to PGE_2_ receptors 2 and 4.^37^ PGF_2α_ is a potent fibrosis factor, which is abundant in the lung bronchoalveolar lavage fluid of patients with idiopathic pulmonary fibrosis.^38^ The fibrotic pathway activated by PGF_2α_ is thought to be independent of the transforming growth factor–β pathway in promoting fibrosis. Similar to PGF_2α_, TXA_2_ also promotes fibrosis, and various TXA_2_ receptor antagonists have been used in antifibrosis studies. The TXA_2_ receptor antagonist NTP42 effectively inhibits inflammatory mast cell infiltration and pulmonary fibrosis and can alleviate experimental pulmonary hypertension.^39^ In patients with pulmonary fibrosis complicated with PAH, PAH promotes the progression of pulmonary fibrosis by exposing capillary ECs to higher mechanical stress.^40^

### Endothelium-derived hyperpolarizing factor

Endothelium-derived hyperpolarizing factor is the third vasodilator, distinct from NO and PGI_2_, produced by ECs. Only arteries with intact ECs exhibit transient hyperpolarization and sustained relaxation in response to acetylcholine stimulation. Endothelium-derived hyperpolarization–mediated vasodilation involves a complex set of electrochemical signaling processes. This mechanism begins with the activation of small (SK_Ca_) or intermediate (IK_Ca_) conductance through Ca^2+^-activated K^+^ channels in ECs by an increased calcium concentration. Then, electric signals are transmitted from hyperpolarized ECs to smooth muscle cells via myoendothelial gap junctions.^1^ Ultimately, this results in smooth muscle cell hyperpolarization and vasodilation. Endothelium-derived hyperpolarizing factors can be divided into epoxyeicosatrienoic acids,^41^ potassium ions,^42^ electric coupling through myoendothelial gap junctions, and others. A recent study suggest that hydrogen sulfide may play an important role in relieving PAH by activating adenosine triphosphate–sensitive potassium channels for vasodilation.^43^

Epoxyeicosatrienoic acids are generated by arachidonic acid catalyzed by cytochrome P450, and Cyp2c9 is a major subtype of cytochrome P450 involved mainly in epoxyeicosatrienoic acid synthesis in ECs.^44^ A recent study showed that 14,15-epoxyeicosatrienoic acid dilates blood vessels by binding to G protein–coupled receptor–39 of vascular smooth muscle cells.^45^ In the proximal pulmonary vessels, ECs rely on releasing PGI_2_ and NO to dilate the vessels. In the distal arterioles, however, ECs rely primarily on endothelium-derived hyperpolarization to dilate the vessels because more myoendothelial gap junctions exist in arterioles than that in proximal arteries.^46^ Myoendothelial gap junctions are composed of connexins, which are responsible for the transport of small molecules between cells and the propagation of electric signals. Connexin 40 is decreased in the lung tissues of experimental pulmonary hypertension models and patients with pulmonary hypertension, and the hypoxia-induced decrease of connexin 40 impairs pulmonary artery relaxation by blocking endothelium-derived hyperpolarization to promote PAH development.^47^

**Figure 3:**
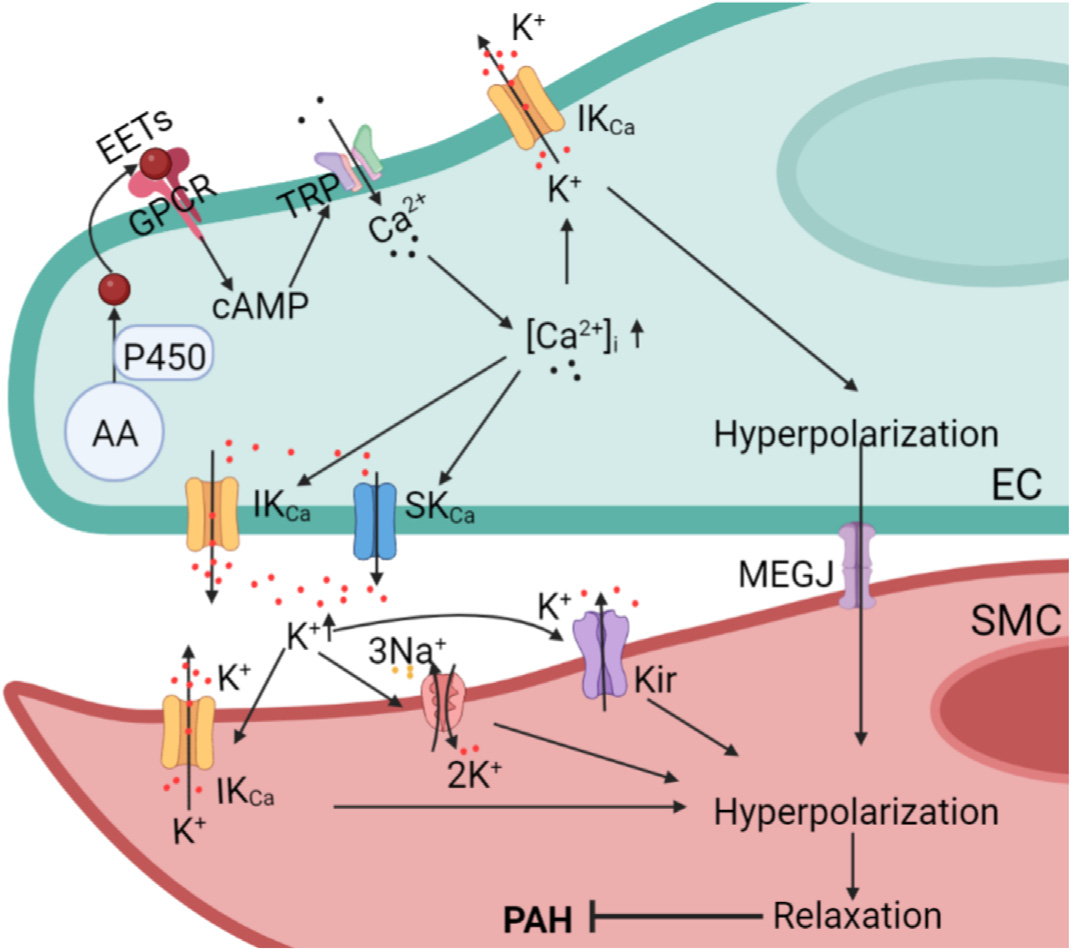
Endothelium-Derived Hyperpolarization Regulates Smooth Muscle Cell Relaxation EDHFs promotes outflow of K^+^ by activating SK_Ca_/IK_Ca_ to form membrane hyperpolarization. High extracellular concentration of K^+^ and myoendothelial gap junctions work together to hyperpolarize SMCs for vasodilation. IK_Ca_ = intermediate conductance through Ca^2+^-activated K^+^ channels; Kir = adenosine triphosphate–sensitive potassium channels; MEGJ = myoendothelial gap junction; P450 = cytochrome P450; SK_Ca_ = small conductance through Ca^2+^-activated K^+^ channels; TRP = transient receptor potential; other abbreviations as in Figures 1 and 2.

It is speculated that there are 2 ways to achieve electric signal transmission from ECs to smooth muscle cells. One is to achieve rapid polarization of smooth muscle cells through myoendothelial gap junctions. The other is through the K^+^ released from ECs into the EC–smooth muscle cell intercellular gap, causing inwardly rectifying K^+^- and Na^+^/K^+^-adenosine triphosphatase–induced hyperpolarization of the smooth muscle cells.^42^ Increased [Ca^2+^]_i_ in ECs is key to endothelium-derived hyperpolarization: studies have shown that activation of endothelial TRPV4 channels triggers uptake of calcium and activates IK_Ca_ and SK_Ca_, promoting vasodilation.^48^ Additionally, PGs, NO, cyclic adenosine monophosphate, and others can affect the concentration of [Ca^2+^]_i_ in ECs. Figure 3 shows the mechanism of endothelium-derived hyperpolarizing factors and endothelium-derived hyperpolarization.

In the progression of PAH, severe remodeling and muscularization of distal pulmonary arterioles causes elevated pulmonary vascular resistance and pulmonary artery pressure. Further study of endothelium-derived hyperpolarization mechanisms may provide a new perspective to elucidate the pathogenesis of PAH, including mechanisms of calcium regulation, the role of conductance Ca^2+^-activated K^+^ channel, functional studies of myoendothelial gap junction components, and development of endothelium-derived hyperpolarization agonists.

### Endothelin-1

ET is a strong vasoconstrictor produced mainly by ECs but is also produced in small quantities by other types of cells, including PASMCs and lung fibroblasts.^49^^,^^50^ Endothelial dysfunction in PAH progression leads to abnormal ET-1 synthesis. There are 3 paralogs for ETs, EDN1, EDN2, and EDN3, encoding ET-1, ET-2, and ET-3, respectively. ET-1 is the most active isoform, with high expression in vascular ECs and vascular smooth muscle cells, airway epithelium, and airway smooth muscle cells.^51^ PreproET-1 is sequentially cleaved by endopeptidase and ET-1-converting enzyme to produce proET-1 and bioactive ET-1.^52^ ET-1 plays an important role in cardiovascular disease because of its biological activity in lung tissue.^53^

ET-1 works primarily through 2 G protein–coupled receptors, ET_A_ and ET_B_, which were first identified in the lung.^54^ Both ET_A_ receptors and ET_B_ receptors mediate vascular smooth muscle cell proliferation, and ET_A_ receptors also mediate vascular contraction. In contrast, ET_B_ receptors on ECs antagonize the contractile effects of ET_A_ by mediating the release of vasodilators and antiproliferative factors and circulating ET-1 clearing.^55^ Although ET_A_ and ET_B_ receptors’ effects are different, the clinical use of ET receptor antagonists is not specifically differentiated. Interestingly, 2 splicing variants of the ET_B_ receptor, ET_B1_ and ET_B2_, perform very different functions,^56^ which are caused by differences in the distribution of the receptor in tissues. ET_A_ and ET_B2_ are present mainly in vascular smooth muscle cells and bind to ET-1 to contract blood vessels,^57^ while ET_B1_ exists mainly in ECs. After binding to ET-1, ET_B1_ promotes the synthesis of NO and PGI_2_ in ECs to antagonize the influence of ET_A_ and ET_B2_ pathways.^58^ A recent study showed that the blood vessels of ET_B_^−/−^ mice were infiltrated by lymphocytes, which contribute to the development of pulmonary hypertension.^59^ However, there remains controversy whether ET_A_ inhibition alone is superior to ET_A_/ET_B_ dual inhibition as therapy for PAH.

There have been many studies on the ET signaling pathway’s role in PAH pathogenesis, and its importance has been supported by many laboratory and clinical studies. There is clear evidence showing that the ET system is activated in almost all preclinical PAH models and in all categories of human pulmonary hypertension.^60^ ET-1 activity is significantly increased in different rat pulmonary hypertension models, including hypoxic pulmonary hypertension rats, monocrotaline-induced pulmonary hypertension rats, and genetically modified pulmonary hypertension rats.^61^, ^62^, ^63^, ^64^ Moreover, the ability to clear ET-1 from circulation was impaired in experimental pulmonary hypertension models and the lungs of patients with pulmonary hypertension.^65^ Plasma and lung ET-1 expression is shown to be positively correlated with the severity of disease in patients with PAH.^66^^,^^67^

### TXA_2_ and 8-iso-PGF_2α_

TXA_2_ is produced by ECs, neutrophils, platelets, and macrophages and is shown to mediate platelet shape change and aggregation, as well as promote smooth muscle contraction and hypertrophy.^68^ TXA_2_ requires TXA_2_ receptor β rather than TXA_2_ receptor α to inhibit vascular endothelial growth factor–induced EC migration, NO production, and angiogenesis.^69^ Activation of phospholipase C-β and inositol triphosphate/diacylglycerol signaling caused by TXA_2_ receptor–Gq coupling completes intracellular calcium mobilization.^70^ TXA_2_-mediated TXA_2_ receptor–G_12/13_ coupling phosphorylates myosin light chain via Rho kinase to affect platelet shape.^71^

It is worth mentioning that 8-iso-PGF_2α_, a PG derivative transformed from esterified arachidonic acid on the cell membrane by free radical attack, contributes significantly to endothelial dysfunction as a TXA_2_ receptor activator.^72^ Through binding and activating the TXA_2_ receptor, 8-iso-PGF_2α_ participates in hypoxia-induced pulmonary hypertension progression.^73^ Additionally, 8-iso-PGF_2α_ caused ET-1 accumulation by activating the TXA_2_ receptor and Rho kinase.^74^ There is in vitro evidence that H_2_O_2_ stimulation can lead to an accumulation of 8-iso-PGF_2α_ in ECs, and the increase of 8-iso-PGF_2α_ in the urine of patients with pulmonary hypertension reflects a lipid peroxidation status as a marker of oxidative stress and inflammation.^75^

Early work in PAH has shown disruption of the balance between TXA_2_ and PGI_2_ in patients,^76^ which reflects endothelial dysfunction as a major cause of platelet activation and persistent pulmonary vasoconstriction.^77^ PGI_2_ achieves its vasodilator and anticoagulation function through PGI_2_ receptor–Gs subunit coupling and cyclic adenosine monophosphate pathway activation. The increase of cyclic adenosine monophosphate in smooth muscle cells rapidly reduces [Ca^2+^]_i_ to achieve vasodilation. Studies have shown that PGI_2_ is similar to PGE_2_ in its association with pain and inflammation.^78^ Peroxisome proliferator–activated receptor α and peroxisome proliferator–activated receptor β/δ activation by PGI_2_ or its analogs can also maintain endothelial function and vasodilation, likely through endothelial NO synthase activation.^79^ PGI_2_ and its analogs have been used in the treatment of PAH because of their powerful effects of reducing platelet aggregation and promoting vasodilation, augmenting cardiac output and pulmonary vascular resistance reduction.^80^

### Renin-angiotensin system

The renin-angiotensin system dominates the homeostatic balance of the cardiovascular system and body fluids. There have been many studies demonstrating that the renin-angiotensin system was involved in PAH development through regulating pulmonary vascular remodeling and pulmonary artery pressure.^81^ The angiotensin-converting enzyme (ACE)–angiotensin II–angiotensin type 1 receptor axis and the ACE2–angiotensin-(1-7)–Mas receptor axis are 2 antagonistic signaling pathways.^82^

Angiotensin II is a linear polypeptide composed of 8 amino acids and is the most important effector in the renin-angiotensin system. After ACE–angiotensin II–angiotensin type 1 receptor axis activation, angiotensin I is converted to angiotensin II by ACE. Angiotensin II binds to 2 receptors: angiotensin type 1 receptor and angiotensin type 2 receptor. Angiotensin II binds to angiotensin type 1 receptor, which promotes vasoconstriction, inflammation, and oxidative stress, while binding to angiotensin type 2 receptor leads to vasodilation.^83^ In recent years, Fried et al^84^ found in studies of nicotine inhalation in mice that angiotensin type 1 receptor–mediated angiotensin II acts on pulmonary blood vessels, leading to increased pulmonary artery pressure and right ventricular hypertrophy. A high-salt diet and high concentrations of angiotensin II can cause pulmonary hypertension with cardiac-renal syndrome.^85^ In contrast, inhibition of angiotensin II expression (using inhibitors and oxygen enrichment) can effectively improve cardiopulmonary function in rodents and relieve pulmonary hypertension symptoms.^86^^,^^87^

ACE2–angiotensin-(1-7)–Mas axis activation plays the opposite role from the ACE–angiotensin II–angiotensin type 1 receptor axis. ACE2 will competitively inhibit the ACE–angiotensin II–angiotensin type 1 receptor axis, converting angiotensin II into angiotensin-(1-7), causing angiotensin-(1-7) to further interact with Mas and counteracting the proliferation, contraction, inflammation, and other phenotypes of pulmonary blood vessels caused by the ACE–angiotensin II–angiotensin type 1 receptor axis.^88^ The overexpression of ACE2 in mice shows a greater resistance to hypoxia and attenuates the development of pulmonary hypertension.^89^

### 5-Hydroxytryptamine

5-HT is both a neurotransmitter in the central nervous system and a vasoconstrictor in the periphery. 5-HT mediates PAH by promoting pulmonary vascular contraction and remodeling. Moreover, 5-HT can induce the proliferation of pulmonary fibroblasts and smooth muscle cells, which contributes to pulmonary vascular remodeling and narrowing of the vessel lumen.^90^^,^^91^ The International Union of Pharmacology classification divides 5-HT receptors into 8 categories: 5-HT1, 5-HT2, 5-HT3, 5-HT4, 5-HT5A/5B, 5-HT6, 5-HT7, and “orphan” receptors.^92^ In experimental pulmonary hypertension, antagonism of 5-HT2B receptors has been therapeutic, and activation of 5-HT2B receptors in bone marrow progenitor cells promotes the development of experimental pulmonary hypertension.^93^^,^^94^ Moreover, 5-HT can affect the balance of oxidative stress in PASMCs by enhancing reactive oxygen species production through Src-related kinase-regulated nicotinamide adenine dinucleotide phosphate oxidase 1 and dysregulated nuclear factor [erythroid-derived 2]–like 2 (Nrf-2) antioxidant mechanisms. In this case, the 5-HT1B receptor is involved in experimental pulmonary hypertension by inducing reactive oxygen species production in the lungs.^95^ In the lamb model of persistent pulmonary hypertension of the newborn, injection of 5-HT increases pulmonary vascular resistance, while injection of the 5-HT2A receptor antagonist ketanserin reduces pulmonary vascular resistance in this experimental model.^96^

### Purinergic signaling in regulation of PAH

Since 1972, numerous studies have demonstrated that adenosine triphosphate acts as an extracellular signaling molecule that controls blood pressure.^97^ In fact, cells including erythrocytes, ECs, and immune cells can produce nucleotides (adenosine triphosphate, adenosine diphosphate, uridine triphosphate, uridine diphosphate) or nucleosides (adenosine) that bind to purinergic receptors for their biological functions. These purinergic receptors contain 2 subfamilies, namely, P1R and P2R. Among them, P1R contains 4 subtypes, and P2R can be further subdivided into 2 branches: P2XRs and P2YRs.^98^ The specific classification of these receptors and their corresponding relationships with ligands are reviewed in detail (Figure 1). After being released into extracellular matrix, nucleosides or nucleotides are regulated by a variety of ectonucleotidases. These are classified into 4 groups of enzymes, including ecto-ATPDase 1, 5′-nucleotidase, nucleotide pyrophosphatase/phosphodiesterase and adenosine deaminase. Adenosine triphosphate, in turn, is metabolized by ecto-ATPDase 1, 5′-nucleotidase, and adenosine deaminase into adenosine monophosphate, adenosine, and inosine. Uridine adenosine tetraphosphate is an EDCF synthesized by vascular endothelial growth factor receptor 2 in vascular ECs, while adenosine is a putative EDRF, and EC-derived adenosine diphosphate can be a putative ECRF because of its ability to activate platelet aggregation. This is due mainly to the different types of receptors they activate. These purinergic receptors mediated signaling events are disrupted during PAH progression. Clinical evidence has shown that plasma adenosine concentrations in patients with PAH are lower than in healthy subjects and that intravenous adenosine can effectively reduce pulmonary artery pressure and right ventricular pressure in patients with pulmonary hypertension.^99^ Experiments in lambs also demonstrate that low doses of adenosine can reduce pulmonary artery pressure by decreasing pulmonary vascular resistance.^100^ In contrast, the contractile effect of uridine adenosine tetraphosphate on the pulmonary artery is accomplished by activating P2YR, which may involve extracellular calcium influx in vascular smooth muscle cell.^101^

### The complex interaction of EDRFs and EDCFs

**Figure 4:**
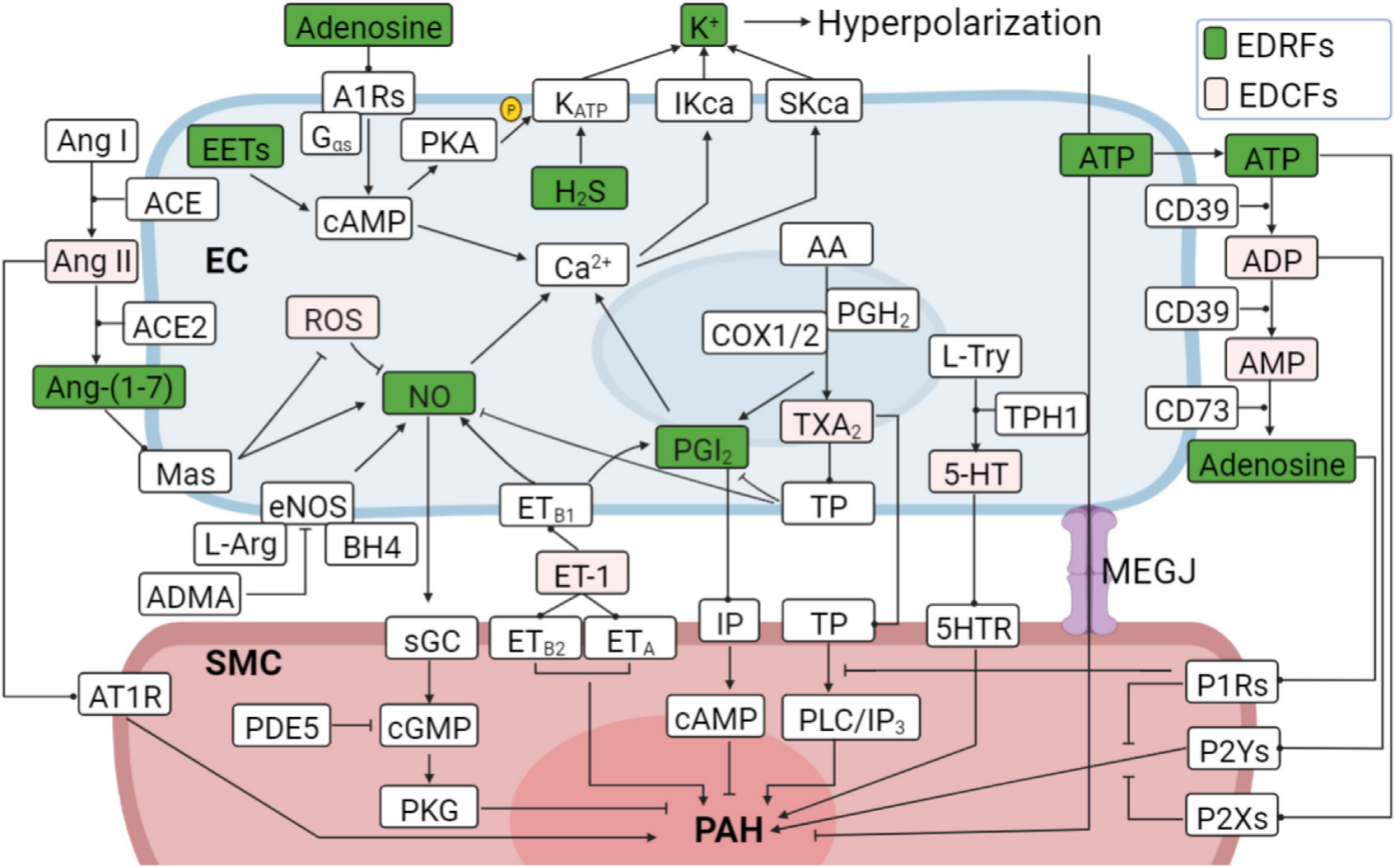
Interactions Among EDRFs and EDCFs Neither EDRFs nor EDCFs function independently, and there are complex interactions between them. ACE = angiotensin-converting enzyme; ADMA = asymmetrical dimethylarginine; BH4 = tetrahydrobiopurine; CD39 = ecto-ATPDase 1; CD73 = 5′-nucleotidase; eNOS = endothelial nitric oxide synthase; L-Arg = L-arginine; PKA = protein kinase A; other abbreviations as in Figures 1 and 2.

The specific function of EDRFs or EDCFs depends on their receptor pathways. There are complex interactions among these factors (Figure 4). ET-1 mediates vasoconstriction by promoting TXA_2_ release and activation of TXA_2_ receptors, which depends on the protein kinase Cα pathway.^102^ The abundance of ET-1 is influenced by several factors, including hypoxia, hyperoxia, reactive oxygen species, growth factors, cytokines, shear stress, thrombin, angiotensin II, and others.^103^^,^^104^ In addition, ET-1 biosynthesis is inhibited by NO and PGI_2_.^105^ The vasodilation function of adenosine can be partially achieved by the activation of the adenosine triphosphate–sensitive potassium channel mediated by the A_2A_Rs–Gαs–protein kinase A pathway.^106^ This process relies on cyclic adenosine monophosphate accumulation and activation of protein kinase A, which phosphorylates the adenosine triphosphate–sensitive potassium channel complex and promotes channel opening. K^+^ efflux induces hyperpolarization of the cell membrane and eventually vascular expansion.^107^ The complex interactions of these opposing networks and the imbalance identified in the pathogenesis of PAH highlight the importance of these pathways and the need for further study.

## Drugs Related to Edrfs and Edcfs for PAH Treatment

Over the past 20 years, the treatment and management of patients with pulmonary hypertension have made numerous advancements in drug development and molecular targeting. Currently, 5 different targeted drugs are available for treatment: ET receptor antagonists, phosphodiesterase-5 inhibitors, soluble guanylate cyclase stimulators, PGI_2_ derivatives, and PGI_2_ agonists. These different classes of drugs are used in various combinations for pulmonary hypertension treatment, and Table 2 highlights the pulmonary hypertension drugs related to EDRFs and EDCFs.

**Table 2:** Drugs Associated With Endothelium-Derived Relaxing Factors and Endothelium-Derived Contracting Factors for PH Treatment

Drug	Target and Function	Indications
Macitentan	ETAR/ETBR antagonist	PAH, CTEPH, IPF
Bosentan	ETAR/ETBR antagonist	PAH, CTEPH, IPF
Ambrisentan	ETAR antagonist	PAH, SSc-PH, IPF
Sitaxsentan^a^	ETAR antagonist	PAH
ETRQβ-002^b^	Vaccine for ETAR	PAH
Beraprost	IP, EP3 receptor agonist	ASO, PAH
Epoprostenol	IP, EP1, EP3 receptor agonist	PAH
Selexipag	IP receptor agonist	PAH
Iloprost	IP, EP1, EP2 receptor agonist	SSc, PAH
Treprostinil	IP, DP1, EP2 receptor agonist	PAH
MRE-269^b^	Selective IP receptor agonist	PAH
Riociguat	Oral stimulator of sGC	PAH, CTEPH
Cinaciguat^b^	sGC activator	PPHN
Tadalafil	PDE-5 inhibitor	PAH
Sildenafil	PDE-5 inhibitor	PAH
Vardenafil^b^	PDE-5 inhibitor	PAH
Inhaled NO^c^	Vasodilator	PAH, PPHN
Inhaled nitrite^b^	Vasodilator	PAH
Oral L-Cit^b^	Intermediate for NO synthesis	PAH
Oral L-Arg^b^	Substrates for NO synthesis	PAH
6R-BH4^b^	Cofactor for eNOS	PAH
Rodatristat ethyl^b^	TPH1 inhibitor	PAH
GSK2586881^b^	Recombinant human ACE2	PAH

ACE2 = angiotensin-converting enzyme 2; ASO = arteriosclerosis obliterans; BH4 = tetrahydrobiopurine; CTEPH = chronic thromboembolic pulmonary hypertension; DP = prostaglandin D_2_ receptor; eNOS = endothelial nitric oxide synthase; EP = prostaglandin E_2_ receptor; ETAR = ET_A_ receptor; ETBR = ET_B_ receptor; IP = prostaglandin I_2_ receptor; IPF = idiopathic pulmonary fibrosis; L-Arg = L-arginine; L-Cit = L-citrulline; PAH = pulmonary arterial hypertension; PDE-5 = phosphodiesterase-5; PH = pulmonary hypertension; PPHN = persistent pulmonary hypertension of the newborn; sGC = soluble guanylate cyclase; SSc = systemic sclerosis; TPH1 = tryptophan hydroxylase 1.

a Sitaxsentan was removed from the market because of liver toxicity.

b Experimental use only. cInhaled NO is for short-term use or experimental use in patients with PAH.

### The therapeutic value of the NO pathway

NO pathway restoration has positive impact on endothelial integrity and is a major target of clinical PAH therapy. The U.S. Food and Drug Administration has approved inhaled NO to treat persistent pulmonary hypertension of the newborn,^108^ which has prompted further development of portable delivery devices and NO inhalation clinical trials for pulmonary hypertension.^16^ Oral L-arginine supplementation effectively increases NO production in patients with pulmonary hypertension and improves hemodynamic status and exercise capacity.^109^ L-citrulline is an intermediate in NO synthesis, and oral L-citrulline supplementation can prevent PAH development.^110^ Endothelial NO synthase gene-enhanced progenitor cells used to treat PAH significantly improved patients’ 6-minute walk distance, but there was no sustained hemodynamic improvement.^111^ Sapropterin dihydrochloride is a tetrahydrobiopurine analog involved NO synthesis that is currently under investigation for treatment of PAH (NCT00435331). Moreover, NO can also be formed from nitrite. A clinical trial demonstrated that nitrite inhalation can relieve pulmonary hypertension symptoms via improvements in left and right ventricular filling pressure and pulmonary artery compliance.^112^ However, the dangers of nitrite overuse have been fully demonstrated. Some recreational or sexual enhancement drugs contain amyl nitrite, and misuse of these drugs can cause serious health damage.

### Targeted drugs based on the NO–soluble guanylate cyclase–cGMP–protein kinase G axis

Many experiments have demonstrated that soluble guanylate cyclase activity is impaired during the development and progression of PAH. The oxidation of the heme group in soluble guanylate cyclase attenuates its response to NO and possibly results in heme’s dissociation from soluble guanylate cyclase.^113^^,^^114^ Significant soluble guanylate cyclase up-regulation was found in pulmonary arteries in patients with idiopathic PAH compared with healthy donors. Additionally, soluble guanylate cyclase was also up-regulated in lungs from hypoxic pulmonary hypertension mice and monocrotaline-induced pulmonary hypertension rats, similar to patients with idiopathic PAH.^115^ On the basis of soluble guanylate cyclase research, 2 new classes of agents have been developed: 1) riociguat, a soluble guanylate cyclase stimulator that activates the native Fe^2+^–soluble guanylate cyclase and synergizes with NO, significantly improving exercise capacity and pulmonary hemodynamic status in patients with pulmonary hypertension, has been approved for treatment PAH^116^; and 2) cinaciguat, a soluble guanylate cyclase activator that activates the Fe^3+^ form, or heme-free form of the enzyme, has been shown to cause pulmonary vasodilation in experimental persistent pulmonary hypertension of the newborn and improve cardiopulmonary hemodynamic parameters in patients with acute decompensated heart failure.^117^^,^^118^

cGMP is metabolized by cGMP-specific 3′,5′-cyclic phosphodiesterase-5 in lung tissue. Phosphodiesterase-5 hydrolyzes the cGMP cyclic phosphate bond to form 5′-guanosine monophosphate, which in turn stimulates protein kinase G. It was reported that phosphodiesterase-5 was elevated both in PASMCs of patients with PAH and cardiomyocytes of patients with right ventricular hypertrophy.^119^^,^^120^ Moreover, phosphodiesterase-5 levels were significantly increased in the pulmonary arteries of hypoxic pulmonary hypertension rats.^121^ Two phosphodiesterase-5 inhibitors, sildenafil and tadalafil, effectively improved 6-minute walk distance and pulmonary hemodynamic parameters in placebo-controlled trials and have been approved by the Food and Drug Administration for clinical treatment of PAH in adults.^108^^,^^122^ In addition, vardenafil, another phosphodiesterase-5 inhibitor, has shown positive effects in PAH but is not approved for use in this population.^123^ Phosphodiesterase-5 inhibitors impair cGMP metabolism, resulting in increased levels of intracellular cGMP, which activates protein kinase G by feedback regulation. Protein kinase G activation causes a series of downstream effects, including vasodilation and inhibition of vascular smooth muscle cell proliferation.^124^ A recent study showed that a novel class of compound, pyrazolo [3,4-b] pyridine derivatives, can not only activate soluble guanylate cyclase to play a vasodilator role but can also regulate vascular remodeling by inhibiting adenosine monophosphate–activated protein kinase.^125^ These compounds appear to show promise but have not been validated clinically.

### PGI_2_ analogues, PGI_2_ receptor agonists, and TXA_2_ inhibitors

PGI_2_ is a very potent vasodilator but is unstable with a short half-life and therefore has limitations to its clinical use and utility. Therapies in this class started as a synthetic analog to PGI_2_, epoprostenol, and have since been modified to retain the potent vasodilatory properties while optimizing pharmacokinetics and pharmacodynamics. These agents include beraprost, iloprost, MRE-269, treprostinil, and selexipag. Treprostinil, a long-half-life PGI_2_ analog, continues to be tested in clinical trials for PAH treatment by intravenous, oral, and inhaled administration.^126^, ^127^, ^128^ Most agonists among them are not specific for PGI_2_ receptor and are involved in inflammatory and immune processes by activating other PG receptors, such as PGE_2_ and PGD_2_ receptor, which may counteract the benefits of PGI_2_ receptor signaling in endothelial maintenance, vasodilation, and anticoagulation.^129^ Selexipag, an oral PGI_2_ receptor agonist, is shown to be highly selective and designed to avoid the effects of PGE_2_ and PGD_2_ receptors.^130^

TXA_2_ antagonists, TXA_2_ receptor antagonists, and thromboxane synthase inhibitors can block TXA_2_-induced platelet aggregation and vasoconstriction to relieve experimental pulmonary hypertension. The thromboxane synthase inhibitor OKY-046 mitigated monocrotaline-induced pulmonary hypertension development by reducing TXA_2_ production in rats,^131^ and similar results were also seen in pulmonary hypertension induced by heparin-protamine complexes in goats.^132^ Additional thromboxane synthase inhibitors include CGS 15435, picotamide, furegrelate sodium, ONO-1301, and others. It is worth mentioning that ONO-1301 is a PGI_2_ analogue that not only activates PGI_2_ receptor in the long term but also inhibits thromboxane synthase activity.^133^ TXA_2_ antagonists include ramatroban and ramatroban-D4, while TXA_2_ receptor antagonists include NTP42, YM158 free base, daltroban, picotamide, ICI 192605, LCB-2853, and others. However, none of these drugs is approved for PAH therapy, or they are undergoing clinical trials.

Predictably, interference in the synthesis of PGs and their receptor pathways may ultimately lead to endothelial dysfunction. Specifically, these include arachidonic acid depletion through lipid peroxidation, dysregulated expression profiles of various PG synthases, and PG receptor activation disorders. Hence, multiple considerations are needed to restore endothelial function. Therefore, we can get a glimpse of future research directions that should focus on the development of highly effective free radical scavengers and specific activators and inhibitors for both PG synthases and PG receptors.

### Clinical therapeutic drugs for pulmonary hypertension based on the ET-1 signaling pathway

Two ET receptor antagonists are currently in clinical use: selective (for ET_A_) and nonselective (both ET_A_ and ET_B_) receptor blockers. Both ET receptor antagonists have been clearly verified to be effective in many preclinical pulmonary hypertension models to improve pulmonary artery EC function, hemodynamic derangements, and right ventricular hypertrophy.^134^, ^135^, ^136^ Currently, some ET receptor antagonists have been approved for PAH clinical therapy: bosentan was the first ET receptor antagonist to have been approved by the Food and Drug Administration in 2001, ambrisentan was approved in 2007, and macitentan was approved in 2013. In addition, sitaxsentan was approved in the European Union, Canada, and Australia, but not in the United States.^51^^,^^137^ It has subsequently been removed from the market because of concerns over liver toxicity.

In general, ET receptor antagonist classification is achieved by differences in pharmacokinetics, basic structure, and receptor affinity, such as bosentan and macitentan being nonselective, while ambrisentan is selective for ET_A_.^137^ In terms of immunotherapy, the first experimental vaccine (ETRQβ-002) against ET_A_ for PAH was recently found. ETRQβ-002 can alleviate remodeling of pulmonary arterioles and the right ventricle in monocrotaline-induced and SU5416/hypoxia-induced pulmonary hypertension models by reducing the pressure response, inhibiting ET-1-initiated signal transduction, and effectively reducing right ventricular systolic pressure.^138^ Whether such immunotherapy is safe and reliable in patients remains to be determined, but vaccination opens new ways to treat PAH.

### Tryptophan hydroxylase 1 is a potential target for PAH therapy

Tryptophan hydroxylase (TPH) catalyzes tryptophan to form serotonin, the rate-limiting step in serotonin synthesis.^139^ There are 2 subtypes of TPHs: TPH1 and TPH2. Previous studies have demonstrated that a portion of 5-HT is produced in pulmonary artery ECs. Endothelium-derived 5-HT promotes PASMC proliferation and PAH development through TPH1. Meanwhile, with PAH developing, 5-HT crosses the intima and contacts PASMCs, causing vasoconstriction, so it is also considered an EDRF. TPH1 is present primarily in the gut and mediates peripheral serotonin production, whereas TPH2 is present exclusively in the central nervous system.^139^ TPH1 expression is increased in the pulmonary artery ECs of patients with PAH and contributes to PASMC hyperplasia,^140^ and endothelial TPH1 expression was also increased in experimental pulmonary hypertension models.^141^ Increasing evidence indicated that TPH1 gene knockout or drug inhibition shows therapeutic effects in experimental pulmonary hypertension models, including the hypoxia-, monocrotaline-, and SU5416/hypoxia-induced rodent pulmonary hypertension models.^142^^,^^143^ Currently, the TPH1 inhibitor rodatristat ethyl (KAR5585) is in the recruitment phase of a clinical trial, and selective inhibitors of TPH1 are expected as new targeted PAH therapies.

### The ACE2-angiotensin-(1-7)–Mas axis antagonizes the effect of angiotensin II in PAH

In recent years, a newly developed oral drug using plant cell encapsulation ACE2/angiotensin-(1-7) reduced the experimental pulmonary hypertension phenotype.^144^ Similarly, a study demonstrated that symptoms in patients with pulmonary hypertension were alleviated when they were given recombinant human ACE2 intravenously.^145^ Most recently, this recombinant protein was developed into a soluble intravenous injection called GSK2586881, which was evaluated for safety and pharmacokinetics in a PAH phase 2 clinic trial.^146^ Moreover, the discovery of the micro–ribonucleic acid let-7b, which targets inhibition of ACE2, augmented the development of experimental pulmonary hypertension and revalidated the cardiovascular protective effect of ACE2.^147^

## Conclusions and Perspectives

The balance of production between EDCFs and EDRFs is a prerequisite for normal vascular function. Healthy pulmonary artery ECs relax blood vessels by releasing NO, PGI_2_, and endothelium-derived hyperpolarizing factors, subsequently reducing pulmonary vascular resistance and pulmonary artery pressure. In pathologic conditions of PAH, or under hypoxia, EDRF production and release are reduced, while EDCFs are increased. This results in leukocyte adhesion, platelet aggregation, PASMC proliferation, and ultimately pulmonary artery contraction and remodeling (Central Illustration). As EDRFs and EDCFs dominate vascular contraction and relaxation, blocking EDCF and activating EDRF signals are good solutions for PAH clinical therapies.

**Central Illustration:**
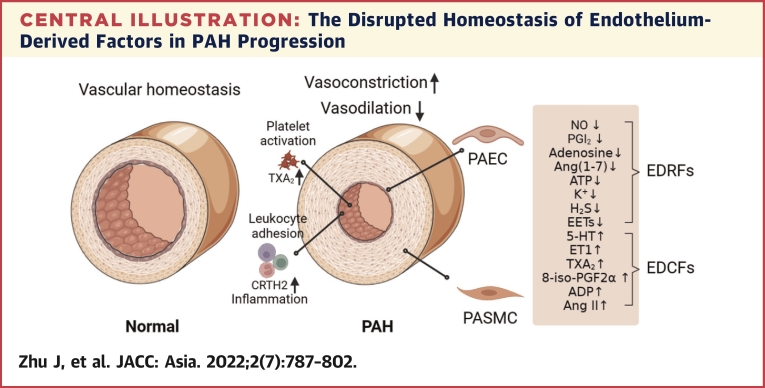
The Disrupted Homeostasis of Endothelium-Derived Factors in PAH Progression During pulmonary arterial hypertension (PAH) development, endothelium-derived relaxing factor (EDRF)/endothelium-derived contracting factor (EDCF) imbalance, platelet aggregation, and leukocyte adhesion all lead to endothelial dysfunction and vasoconstriction. 5-HT = 5-hydroxytryptamine; ADP = adenosine diphosphate; Ang = angiotensin; ATP = adenosine triphosphate; CRTH2 = prostaglandin D_2_ receptor 2; EET = epoxyeicosatrienoic acid; ET = endothelin; PAEC = pulmonary artery endothelial cell; PASMC = pulmonary artery smooth muscle cell; PG = prostaglandin; TXA_2_ = thromboxane A_2_.

Clinical treatment for PAH is still a serious challenge. Often a single drug falls short of controlling severe PAH, and combination therapy with targeted drugs has become an attractive option and standard of care.^148^ Combination therapy with 2 oral pulmonary vasodilators, tadalafil and ambrisentan, was the first to prove the benefit of early treatment targeting multiple pathogenic molecular pathways.^149^ Since that time multiple studies have shown the benefit of combination therapy and the positive effects of adding additional agents to background therapy for patients with PAH.^150^ In addition to dual therapy, the therapeutic effects of upfront triple therapy have received attention. A randomized controlled trial study in 2021 included patients with different subtypes of pulmonary hypertension (123 receiving initial triple therapy vs 124 receiving initial dual therapy).^151^ It is undeniable that the combination of targeted drugs is generally superior to a single drug in PAH therapy. However, there is an urgent need to explore more therapeutic targets and targeted drugs. Optimization on the basis of the combination of multiple targeted drugs may eventually provide an effective solution.

## Funding Support and Author Disclosures

This work was funded in part by the National Key Research and Development Program of China (grant 2019YFE0119400), the Natural Science Foundation of China (grants 81970052 and 82170057), and the National Lung, Heart, and Blood Institute of the National Institutes of Health (grant R35 HL135807). The authors have reported that they have no relationships relevant to the contents of this paper to disclose.
